# Influence of sinker timing on loop shape, width and areal density of weft-knitted cotton plain jersey fabric

**DOI:** 10.1371/journal.pone.0323572

**Published:** 2025-05-15

**Authors:** Mohammad Hossain, AKM Mobarok Hossain, Md. Abdullah Al. Mamun, Aiead Ibne Fahim, Nowshin Nigar Rafa, Emtiaz Ahmed Pappu, Abu Darda, Shafin Arafat, Syed Tohedul Islam

**Affiliations:** 1 Department of Fabric Engineering, Bangladesh University of Textiles (BUTEX), Dhaka, Bangladesh; 2 Department of Textile Engineering, BGMEA University of Fashion and Technology (BUFT), Dhaka, Bangladesh; 3 Department of Textile Engineering, Ahsanullah University of Science and Technology (AUST), Dhaka, Bangladesh; Coventry University, UNITED KINGDOM OF GREAT BRITAIN AND NORTHERN IRELAND

## Abstract

This study aims to explore the influence of sinker timing—a relative positional setting of two primary knitting elements, i.e., needle and sinker, on some important knitted fabric parameters and related properties. Plain jersey fabric samples were produced from cotton yarn (linear density of 19.68 Tex) at three different quality values (loop lengths of 2.77 mm, 2.84 mm, and 2.90 mm respectively) on a positive feed-based multi-feeder circular knitting machine. Three different sinker timings (regular, forwarding, and retracting) were used for each quality setting; thus, a total of 9 (nine) fabric samples were developed for experimental purposes. It was found that forward sinker timing resulted in an increase in the loop shape factor concerning regular sinker timing and vice versa. However, stitch densities were almost the same for all settings of sinker timing at a particular value of loop length. Consequently, fabric width was highest for forward timing and fabric areal density remained almost unchanged. Also visual inspection revealed no noticeable differences among the fabric samples.

## Introduction

Knitting is the second most prevalent method of fabric production, which contributes to around 20 percent of global fabric production. Particularly weft-knit sector contributes mostly to the world market of knitted fabrics [1], comprising both single-jersey and double-jersey structures. Weft-knitted Single Jersey is a versatile and popular type of knitted fabric, made by interlocking a series of loops in a single row. This group of fabrics is favored worldwide for its lightweight, softness, and stretchability [2]. As per Volza’s Global Export data, Single jersey fabric (specifically HSN Code 60069000) export shipments stood from the World at 9700 with 543 exporters and 600 buyers [3].

Most single-jersey fabrics are produced on circular machines (often referred to as ‘Sinker Top Machine’) in which a set of needles, mounted vertically in a needle cylinder, co-operates with a corresponding set of horizontal sinkers mounted radially in a sinker ring [4]. The global Single Jersey Circular Knitting Machines market was valued at US$ 323.6 million in 2023 and is anticipated to reach US$ 404.9 million by 2030 indicating a growing demand from fabric manufacturers at present [5].

Circular knitting Machine developers worked over the years to upgrade knitting machine features, including machine gauge, machine speed, yarn feeding system, knitting elements, and more. These parameters play a significant role in determining the quality of knit fabric, including fabric width, areal density, dimensional stability, and mechanical properties like pilling resistance and bursting strength. Therefore, maintaining the machine parameters in balance is the main concern of a knitter or industry practitioner while producing high-quality fabric [6]. Many researchers worked on improving the look and physical qualities of knit fabrics, by changing different knitting and machine parameters. Abedin et al. [7] studied the influence of circular weft knitting machine gauge variation on the mechanical and physical characteristics of 100% cotton knit fabrics and discovered that the fabric areal density, fabric width, dimensional stability, and bursting strength had remarkably changed due to gauge variation. Ovi et al. [8] investigated the impact of machine diameter and gauge, finished cloth width, and stitch length on basic knitted structures. Hossain et al. [9] studied the impact of machine speed on loop length and yarn tension. Researches were also carried out to investigate the influence of stitch length [10–12], yarn characteristics [13–17], or spinning system [18], or fabric design [19–21] on various fabric properties. Nevertheless, a research gap can be identified till now where sinker setting, particularly, sinker timing has not been studied as a variable contributing to fabric properties. Sinker timing refers to the relative positioning of sinkers with the needles when sinkers are on the Push Point [22]. This study aims to analyze the impact of sinker timing (categorized as regular, forwarding, and retracting [23] on basic fabric characteristics like loop shape, width, and areal density. Also, fabric appearance will be visually evaluated due to changes in sinker timing. Thus this study will advance the knowledge of sinker timing.

### Significance of the study

**Fig 1:**
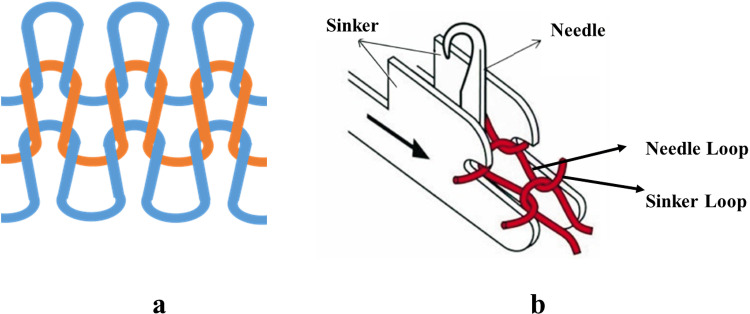
a) Knitted loops in a weft-knitted structure b) Needle loop and sinker loop [24].

Sinker is the most important element of the knitting machine after needles. This element’s basic functions are to act as a web holder and to provide support for knock-over during the knitting process [22]. Sinker timing indicates a mechanical adjustment where the shape or size of a sinker loop (Fig 1) is altered by adjusting a sinker’s relative movement to the needle.

Earlier studies [25] showed that loop length (length of yarn in a knitted loop) and loop shape significantly influence fabric dimension and areal density. However, the adjustment in such loop characteristics during the knitting operation is commonly carried out by controlling the yarn feed system which results in multiple complex changes [26] in different dimensional and quality attributes of the fabrics. As sinker timing brings some changes in loop characteristics also, there are some scopes to study its influence on fabric characteristics and appearance, which may be an extra tool for a knitter to engineer the knitted fabric.

## Materials and methods

### Materials

A laboratory-based sinker top knitting machine was deployed to produce plain jersey fabric samples. The machine specification and yarn parameters are shown in Table 1.

**Table 1:** Machine specification and yarn parameters.

Machine Specifications		Yarn Specifications	
Machine Diameter x Gauge	10 inch (25.4 cm) × 24 Gauge	Yarn type	100% cotton combed, single ply
Type	Single cylinder Latch needle weft knitting machine	Count (Nominal)	30/1 Ne (19.68 Tex)
No. of feeders	30	Count (Actual)	19.78 Tex (s = 0.03)
No. of needles	744	Twist per inch (Nominal)	20
Yarn feeding system	Positive	Twist per inch (Actual)	19.6 (s = 0.5)
No. of the cam track	4	Twist direction	Z

### Sample production

**Fig 2:**
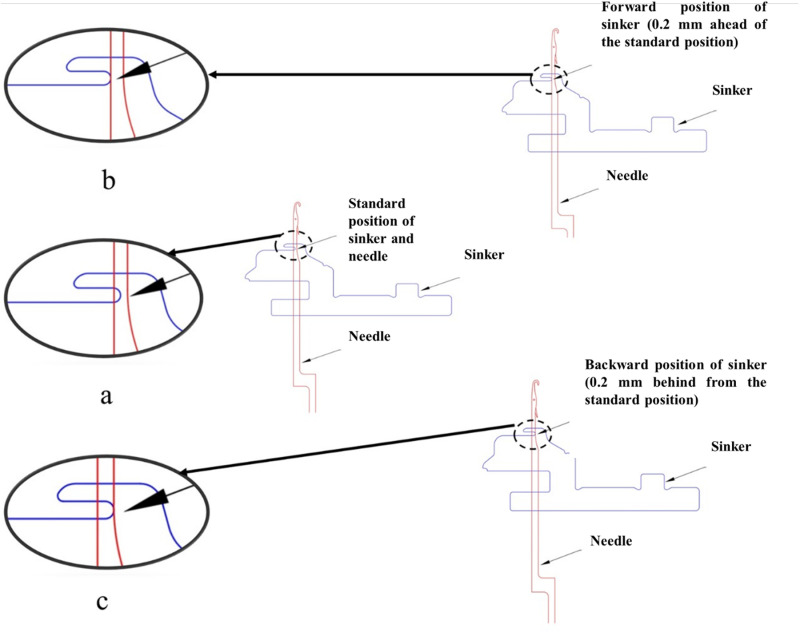
a) Regular, b) Forwarding, and c) Retracting sinker timing.

As the key concern of this research is to explore the influence of sinker timing, plain single jersey fabric samples were produced by varying sinker timing while keeping other parameters constant. This was done for three values of course lengths, which were adjusted using the quality pulley of the positive feed system. At first, the highest and lowest tolerance limits for the sinker cam ring to move back and forth were determined through the control screw. Afterward, three distinguishable sinker timing settings were identified on the experimental machine through a dial indicator where fabric quality (considering fabric defects) remained unaffected. These settings were marked as regular, forwarding (sinkers’ advancement is 0.2 mm from the regular; measured by a dial indicator), and retracting (sinkers’ retraction is 0.2 mm from the regular, measured by a dial indicator) which were adjusted on the machine by changing the position of the adjusting bolt of the sinker cam boxes located on the sinker cam ring. These different sinker timings are illustrated in Fig 2.

**Fig 3:**
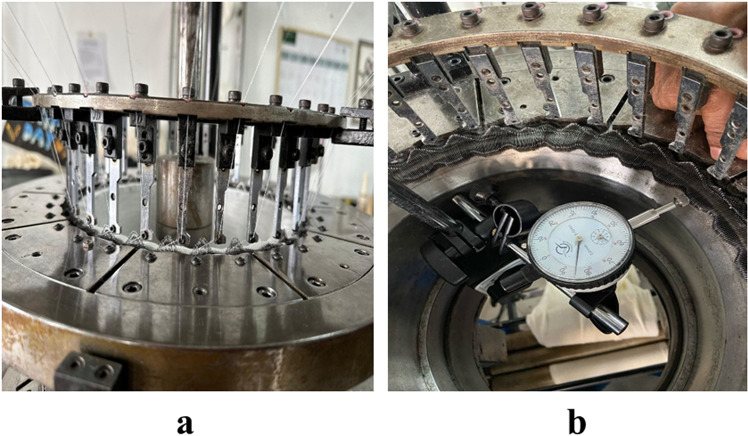
a) Knitting machine head and b) Sinker timing adjustment through a dial indicator.

A total of 90 m of fabric was produced containing 9 different fabric samples (around 10 m of each). The knitting machine head and dial indicator (for adjustment of sinker timing) while producing the samples are shown in Fig 3.

### Measurement of fabric parameters

After knitting, the fabric samples were taken off the knitting machine and dry-relaxed for 48 hours (temperature: 22° C, relative humidity: 67%). Each relaxed sample was cut into three parts. The first part was for the measurement of fabric loop length and the second part was for counting courses per unit length and wales per unit width. The third part was kept for actual measurements of fabric width and areal density (to verify calculated width and areal density). A HATRA course length tester was used to measure course length and subsequently loop length (loop length is equal to course length divided by the number of needles knitting) following BS 5441:1988 [27]. Course and Wale densities were measured through a counting glass following the same standard. Consequently, loop shape factor, fabric width, areal density, and width-wise contraction% were calculated using equation (i) [25], equation (ii) [25], equation (iii) [27], and equation (iv) [20].


$$Loop\;shape\;factor\;\;=\;\frac{Number\;of\;courses\;per\;unit\;length}{Number\;\;of\;walse\;per\;unit\;width}$$ (i)

$$Fabric\;width\;(cm)\;=\;\frac{Number\;of\;wales\;or\;number\;\;of\;active\;needles}{Number\;of\;wales\;per\;cm}$$ (ii)

$$Areal\;density\;(grams\;per\;square\;meter)\;=\;\frac{Number\;\;of\;loops\;\;per\;square\;cm\;x\;loop\;length\;(mm)\;x\;yarn\;linear\;density\;(Tex)}{100}$$ (iii)

$$Width-wise\;contraction\;\backslash \%\;=\;(\frac{Number\;of\;wales\;per\;2.54\;cm-Machine\;gauge}{Number\;of\;wales\;per\;2.54\;cm}\times 100)$$ (iv)

All the obtained values are displayed in Table 2.

**Table 2:** Loop shape factor, fabric width, and areal density for different sinker timing.

Sample number	Sinker timing	Loop length (mm)	Wales per 2.54 cm	Courses per 2.54 cm	Loop shape factor	Stitch density (number of loops per [2.54 cm]^2^)	Fabric width (cm)	Fabric areal density (GSM)
1	Regular	2.77	32.2 (s = 0.42)	47.8 (s = 0.42)	1.48	1539.16	58.70	130.1
2	Regular	2.84	30.8 (s = 0.42)	45.9 (s = 0.32)	1.49	1413.72	61.37	122.5
3	Regular	2.90	29.3 (s = 0.48)	43.8 (s = 0.42)	1.49	1283.34	64.49	113.5
4	Retracting	2.77	33.5 (s = 0.53)	45.5 (s = 0.53)	1.36	1524.25	56.41	128.8
5	Retracting	2.84	32 (s = 0.47)	43.9 (s = 0.32)	1.37	1404.80	59.06	121.7
6	Retracting	2.90	30.4 (s = 0.84)	42.1 (s = 0.32)	1.38	1279.84	62.15	113.2
7	Forwarding	2.77	31.3 (s = 0.48)	49 (s = 0.67)	1.57	1533.70	60.38	129.6
8	Forwarding	2.84	29.8 (s = 0.42)	47.5 (s = 0.53)	1.59	1415.5	63.42	122.6
9	Forwarding	2.90	28.3 (s = 0.48)	45.1 (s = 0.32)	1.59	1276.33	66.78	112.9

s = standard deviation.

## Results and discussion

### Loop shape factor

Loop shape factor is the ratio of the courses per unit length to the wales per unit width and thus it is a determinant for fabric dimensions. Loop shape factor also influences fabric performance like spirality [28].

**Fig 4:**
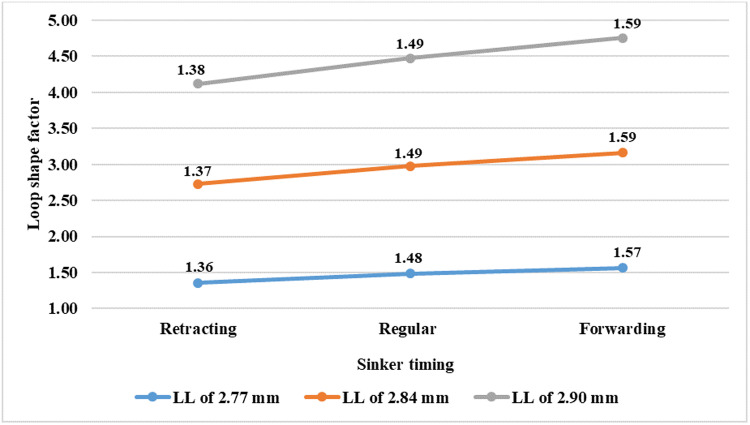
Loop shape factor at different sinker timings.

Fig 4 shows that forward timing results highest value of the loop shape factor whereas retracting timing results in the lowest value.

**Fig 5:**
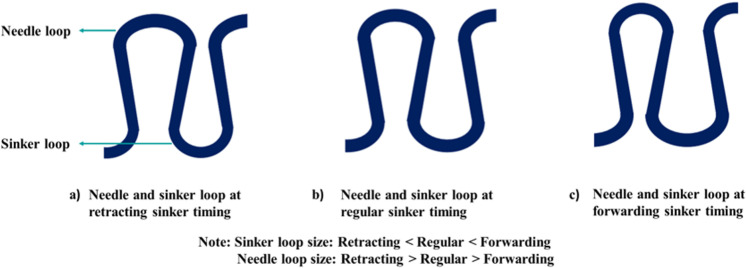
Schematic diagrams of loop shapes at various sinker timings.

This is true for all values of loop lengths (LL). It may be attributed to the fact that in forward sinker timing, the sinker pushes more forward and robs back more yarn from two neighboring needle loops than the regular timing. This results in a comparatively larger sinker loop and smaller needle loop than regular timing. On the contrary, retracting sinker timing results in a comparatively smaller sinker loop and larger needle loop than regular timing. These have been shown in Fig 5.

**Fig 6:**
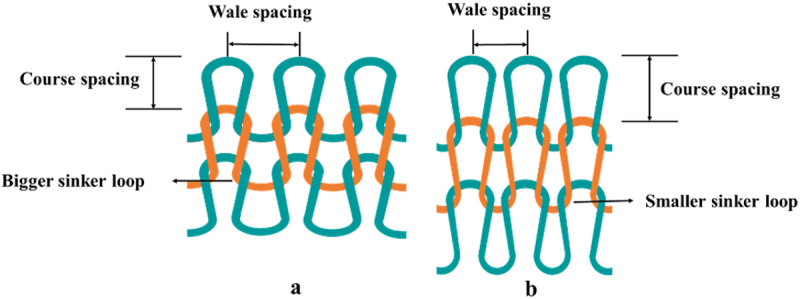
Wale and course spacing at a) forwarding and b) retracting sinker timing.

Hence, forwarding sinker timing increases wale spacing, reduces course spacing (illustrated in Fig 6), and therefore enhances the loop shape factor compared to the regular timing. On the contrary, retracting timing lessens the loop shape factor upon fabric relaxation. Thus, the loop shape is significantly influenced by the sinker timing.

**Fig 7:**
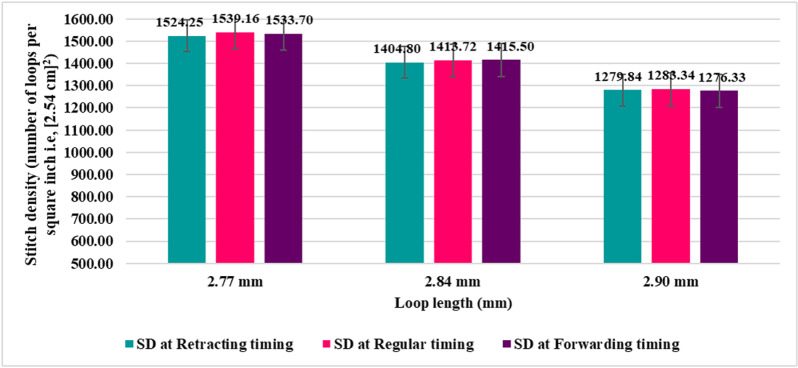
Stitch density at different sinker timings.

However, as the change (reduction or increase) of wale spacing was accompanied by an increase or a reduction of course spacing, stitch density (SD) values were not significantly different for all variations of sinker timings, which may be found in Fig 7.

### Fabric width

Fabric width (FW) at the grey relaxed state is an important dimensional parameter and it may be used later to predict finished relaxed fabric width with the help of the finishing factor [19]. Thus precision adjustment of fabric width during knitting helps in controlling finished fabric width and hence increases marker efficiency during garment manufacturing. Fabric width is generally calculated using Equation (ii).

**Fig 8:**
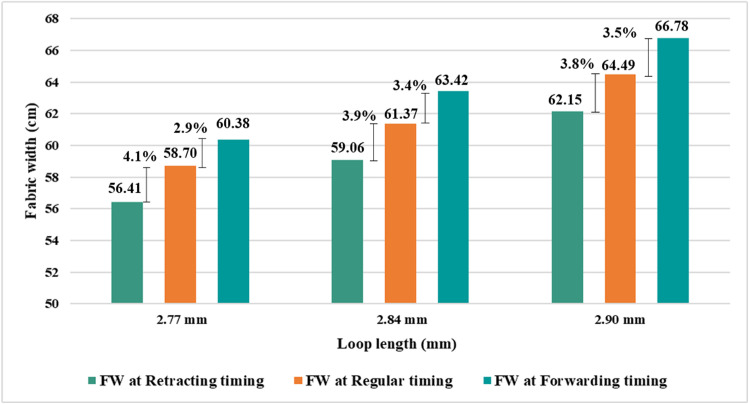
Fabric width variation at different sinker timings.

From Fig 8 it can be seen that fabric width increases for forwarding sinker timing and decreases for retracting sinker timing compared to regular timing while knitting with the same knitting parameters. In particular, there was around a 3.9% decrease in width for retracting sinker timing and a 2.9% increase in width for forwarding sinker timing at a loop length value of 2.77 mm compared to regular sinker timing. Similar trends were also observed for other loop length values (2.84 mm and 2.90 mm). Therefore, it is possible to bring some width adjustment through sinker timing while knitting at the same quality.

### Test of significance

Two sample t-tests were conducted to determine whether the width variations due to different settings of sinker timing were statistically significant. Since fabric width is directly dependent on wale density [according to Equation (ii)], the test of significance was carried out for wale densities between two different timings for a particular fabric quality, i.e., loop length. The null hypothesis had been assumed as there was no statistically significant difference between the sample sets. For example, one of the null hypotheses was that there was no statistically significant difference between the wale densities obtained at a loop length value of 2.77 mm for retracting and regular sinker timing. Thus, a total of 9 (nine) t-tests were conducted, and the obtained results are summarized in Table 3.

**Table 3:** Results of t-test for wale density.

Loop length (mm)	Sample set	No. of observations for wales/2.54 cm	Mean difference between wale densities	Critical value (as obtained from a two-tailed t-distribution table) [29]	t-value	p-value
2.77	Retracting-Regular	10 + 10	1.3	2.10	6.07	0.0001859
	Regular-Forwarding	10 + 10	0.9	2.10	4.45	0.001600197
	Retracting-Forwarding	10 + 10	2.2	2.10	4.31	0.001961689
2.84	Retracting-Regular	10 + 10	1.2	2.10	6	0.000202499
	Regular-Forwarding	10 + 10	1	2.10	5.26	0.000520734
	Retracting-Forwarding	10 + 10	2.2	2.10	11	0.00000160993
2.90	Retracting-Regular	10 + 10	1.1	2.10	3.55	0.006215892
	Regular-Forwarding	10 + 10	1	2.10	4.76	0.001029671
	Retracting-Forwarding	10 + 10	2.1	2.10	6.77	0.0000817769

From Table 3, it can be observed that the t-value (t-statistic) is higher than the critical value in every case. The obtained t-value thus rejects the null hypothesis and it indicates that there is a statistically significant difference between the wale densities and (therefore, fabric widths) of two different timings of a particular quality based on the 95% confidence level.

### Width contraction

Fabric width contraction refers to the width reduction of grey relaxed fabric concerning needle bed dimension which may be expressed as Equation (iv). This knowledge helps a knitter estimate the grey fabric width prior to knitting for a particular machine specification. Thus, width contraction also represents a part of the dimensional stability for a knitted fabric.

**Fig 9:**
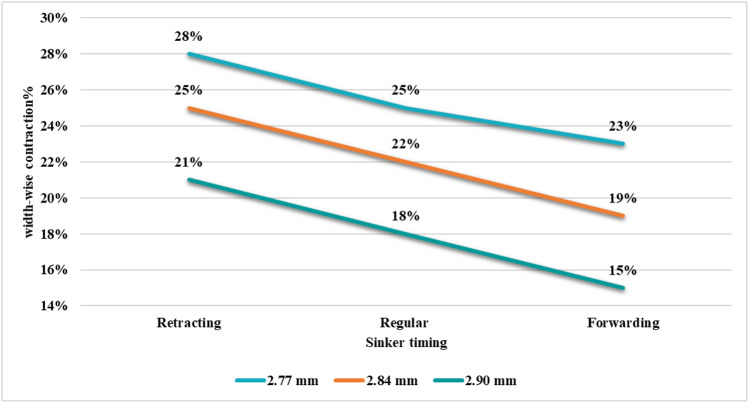
Width-wise contraction% at different sinker timings.

From Fig 9 it can be realized that there is an impact of sinker timing on fabric width contraction. Among three sinker timings, retracting sinker timing caused the highest width contraction and forwarding timing caused the lowest width contraction while knitting parameters were constant. Particularly, at 2.77 mm loop length, width contraction was found as 3% more for retracting sinker timing and 2% less for forwarding sinker timing than that of regular sinker timing. Nearly similar patterns were also observed for fabrics knitted at loop lengths of 2.84 mm and 2.90 mm. Thus, width contraction, as a contributor to a specific fabric width, is also correlated to sinker timing.

### Areal density

Fabric areal density (AD) is an essential physical characteristic associated with comfort, dimensional stability, and many other properties*.* It is generally expressed by the GSM (Grams per square meter) number which represents the mass of a fabric piece in grams that measures over one square meter.

**Fig 10:**
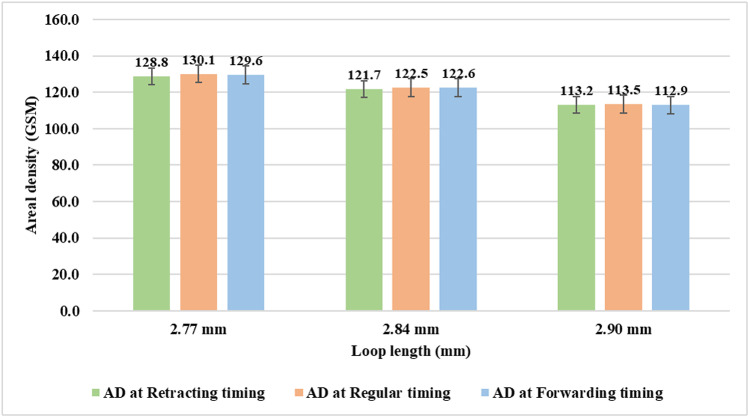
Areal density at different sinker timings.

From Fig 10 it may be found that variation in sinker timing brings no noticeable change in areal density at a particular loop length value. This may be attributed to the fact that the variables in Equation (iii) for determining areal density are either independent (loop length and yarn count) or almost unaffected (stitch density) by the change in sinker timing at a particular loop length value.

### Fabric appearance

**Fig 11:**
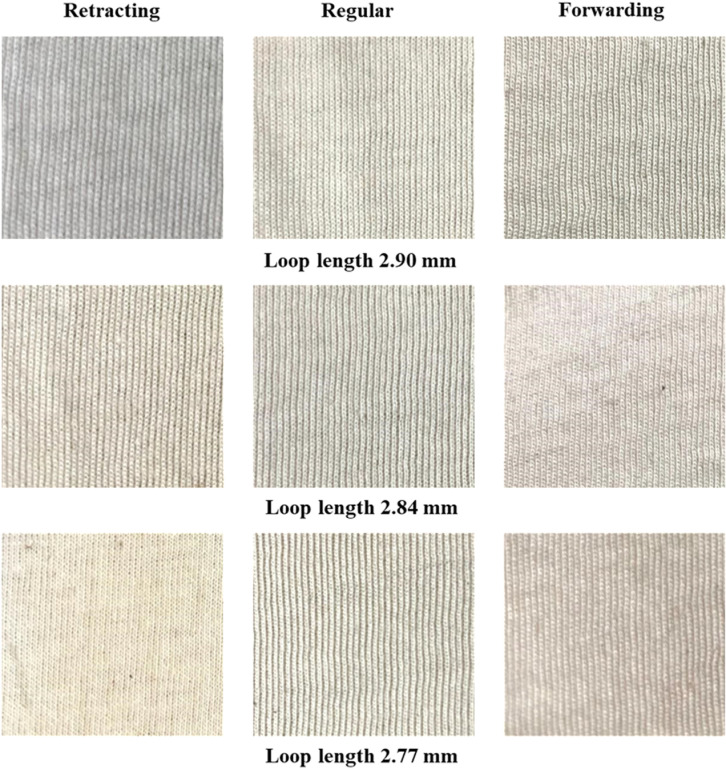
Technical face of the experimental samples.

**Fig 12:**
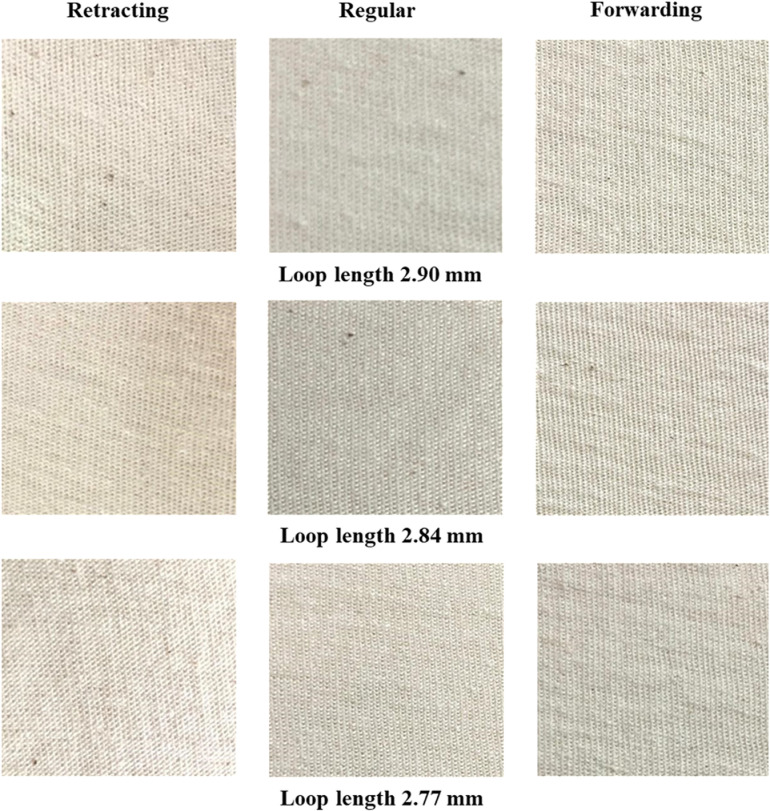
Technical back of the experimental samples.

Fabric samples were subjected to D-65 light which is normally used in the knitting industry for grey fabric inspection. On visual assessment, no significant differences in fabric appearance were observed. The images of the samples are also presented in Fig 11 (technical face) and Fig 12 (technical back). On the whole, various sinker timings bring no noticeable change in fabric appearance.

## Conclusion

Knitted fabric manufacturers generally adjust common knitting variables like yarn count, loop length, or machine type (i.e., machine gauge and diameter) for knitting fabrics at a required quality like GSM, or dimension like width. This research explored the scope of a mechanical adjustment of the knitting machine, i.e., sinker timing to do the same for a knitter. The results showed that sinker timing has no noticeable influence on stitch density, or the areal density of the fabric knitted with the same knitting variables. However, changes in sinker timing affected the loop shape factor and ultimately fabric dimensions like width. This is a noteworthy finding as it gives the knitter an auxiliary tool to adjust fabric dimensions somewhat without altering fabric quality or appearance. In particular, around 7% differences were found in fabric widths between two experimental fabric samples knitted with the same knitting variables but at two extreme points (retracting and forwarding) of sinker timing. Consequently, around a 5% difference was found in width contraction between these samples. Modern industrial knitting machines may possess more capacity for width adjustments through sinker timing variations. Therefore, this particular machine setting, i.e., sinker timing may act as a special tool for fabric width adjustment. Such an adjustment will turn into increased marker efficiency by reducing fabric wastage in the cutting section afterwards. Hence, the implementation of the findings of this research in a knitting industry aligns to achieve the Sustainable Development Goal (SDG). Further research works may be carried out to evaluate fabric behaviors like shrinkage or skewness that are particularly dependent on loop shape, due to changes in sinker timing.

## Supporting information

S1 File **S1 Table.** Raw data on yarn count and twist per inch (TPI) for mean and standard deviation. S = Standard deviation. **S2 Table.** Wale and course density data at 2.77 mm loop length for mean and standard deviation. S = Standard deviation. **S3 Table.** Wale and course density data at 2.84 mm loop length for mean and standard deviation. S = Standard deviation. **S4 Table.** Wale and course density data at 2.90 mm loop length for mean and standard deviation. S = Standard deviation.
(DOCX)
