# Supplementary material for: Influence of sinker timing on loop shape, width and areal density of weft-knitted cotton plain jersey fabric
Source: PLoS One. 2025 May 15;20(5):e0323572. doi: 10.1371/journal.pone.0323572 (PMC12080846; doi:10.1371/journal.pone.0323572)
Supplement: S1 File — S1 Table. Raw data on yarn count and twist per inch (TPI) for mean and standard deviation. S = Standard deviation. S2 Table. Wale and course density data at 2.77 mm loop length for mean and standard deviation. S = Standard deviation. S3 Table. Wale and course density data at 2.84 mm loop length for mean and standard deviation. S = Standard deviation. S4 Table. Wale and course density data at 2.90 mm loop length for mean and standard deviation. S = Standard deviation. (DOCX) [file pone.0323572.s001.docx]

**S1 Table. Raw Data on yarn count and TPI for mean and standard deviation**

| **Yarn count (Tex)** | **TPI** |
| --- | --- |
| 19.79 | 20 |
| 19.82 | 19 |
| 19.75 | 20 |
| 19.75 | 19 |
| 19.79 | 20 |
| 19.78 | 20 |
| 19.82 | 20 |
| Mean = 19.78 | Mean = 19.6 |
| S = 0.03 | S = 0.5 |

S = Standard deviation

**S2 Table. Wale and course density data at 2.77 mm loop length for mean and standard deviation**

| **Retracting sinker timing** | | **Regular sinker timing** | | **Forwarding sinker timing** | |
| --- | --- | --- | --- | --- | --- |
| **Course per 2.54 cm** | **Wale per 2.54 cm** | **Course per 2.54 cm** | **Wale per 2.54 cm** | **Course per 2.54 cm** | **Wale per 2.54 cm** |
| 45 | 34 | 48 | 32 | 49 | 31 |
| 46 | 34 | 48 | 32 | 50 | 31 |
| 45 | 33 | 48 | 32 | 49 | 31 |
| 45 | 34 | 47 | 33 | 48 | 32 |
| 46 | 33 | 48 | 32 | 49 | 32 |
| 46 | 33 | 48 | 32 | 49 | 31 |
| 45 | 33 | 48 | 33 | 49 | 31 |
| 45 | 34 | 47 | 32 | 50 | 31 |
| 46 | 34 | 48 | 32 | 49 | 32 |
| Mean = 45.5 | Mean = 33.5 | Mean = 47.8 | Mean = 32.2 | Mean = 49 | Mean = 31.3 |
| S = 0.53 | S = 0.53 | S = 0.42 | S = 0.42 | S = 0.67 | S = 0.48 |

S = Standard deviation

**S3 Table. Wale and course density data at 2.84 mm loop length for mean and standard deviation**

| **Retracting sinker timing** | | **Regular sinker timing** | | **Forwarding sinker timing** | |
| --- | --- | --- | --- | --- | --- |
| **Course per 2.54 cm** | **Wale per 2.54 cm** | **Course per 2.54 cm** | **Wale per 2.54 cm** | **Course per 2.54 cm** | **Wale per 2.54 cm** |
| 44 | 32 | 46 | 30 | 48 | 30 |
| 44 | 32 | 46 | 31 | 47 | 30 |
| 44 | 32 | 46 | 31 | 48 | 29 |
| 44 | 32 | 46 | 31 | 48 | 30 |
| 43 | 33 | 46 | 31 | 47 | 30 |
| 44 | 32 | 45 | 31 | 47 | 30 |
| 44 | 32 | 46 | 30 | 48 | 30 |
| 44 | 32 | 46 | 31 | 47 | 30 |
| 44 | 32 | 46 | 31 | 48 | 30 |
| Mean = 43.9 | Mean = 32 | Mean = 45.9 | Mean = 30.8 | Mean = 47.5 | Mean = 29.8 |
| S = 0.32 | S = 0.47 | S = 0.32 | S = 0.42 | S = 0.53 | S = 0.42 |

S = Standard deviation

**S4 Table. Wale and course density data at 2.90 mm loop length for mean and standard deviation**

| **Retracting sinker timing** | | **Regular sinker timing** | | **Forwarding sinker timing** | |
| --- | --- | --- | --- | --- | --- |
| **Course per 2.54 cm** | **Wale per 2.54 cm** | **Course per 2.54 cm** | **Wale per 2.54 cm** | **Course per 2.54 cm** | **Wale per 2.54 cm** |
| 42 | 30 | 44 | 30 | 46 | 28 |
| 42 | 32 | 44 | 29 | 45 | 28 |
| 42 | 30 | 43 | 29 | 45 | 28 |
| 42 | 30 | 44 | 30 | 45 | 29 |
| 42 | 30 | 44 | 29 | 45 | 28 |
| 42 | 30 | 44 | 29 | 45 | 28 |
| 42 | 32 | 44 | 29 | 45 | 29 |
| 43 | 30 | 44 | 29 | 45 | 29 |
| 42 | 30 | 43 | 29 | 45 | 28 |
| Mean = 42.1 | Mean = 30.4 | Mean = 43.8 | Mean = 29.3 | Mean = 45.1 | Mean = 28.3 |
| S = 0.32 | S = 0.84 | S = 0.42 | S = 0.48 | S = 0.32 | S = 0.48 |

S = Standard deviation
